# Propensity-Matched Analysis of Del Nido Cardioplegia in Adults Undergoing Cardiac Surgery with Prolonged Cross-Clamping Time

**DOI:** 10.21470/1678-9741-2020-0309

**Published:** 2022

**Authors:** Utkan Sevuk, Seyithan Dursun, Elif Sevgi Ar

**Affiliations:** 1 Department of Cardiovascular Surgery, Diyarbakir Gazi Yasargil Training and Research Hospital, Diyarbakir, Turkey.; 2 Department of Perfusion, Diyarbakir Gazi Yasargil Training and Research Hospital, Diyarbakir, Turkey.

**Keywords:** Myocardial Protection, Cardiopulmonary Bypass, Cardiac Surgery, Heart Arrest, Induced, Intra-Aortic Ballon Pumping

## Abstract

**Introduction:**

There is not enough data in the literature regarding the safety and efficiency of del Nido cardioplegia in patients with prolonged cross-clamping time. This study aims to determine the efficacy and safety of del Nido cardioplegia compared to cold blood cardioplegia in patients with prolonged aortic cross-clamping time.

**Methods:**

In this retrospective study, patients with an aortic cross-clamping time ≥ 90 minutes were included. One hundred consecutive adult patients undergoing cardiac surgery using del Nido cardioplegia comprised the study group, and 100 consecutive adult patients undergoing cardiac surgical procedures using cold blood cardioplegia comprised the control group. Propensity score matching yielded 88 del Nido cardioplegia and 88 cold blood cardioplegia patients.

**Results:**

There were no significant differences when comparing the matched groups regarding the requirement for intraoperative defibrillation, postoperative peak troponin T levels, inotropic support, intra-aortic balloon pump requirement, and left ventricular ejection fraction at discharge and on the sixth postoperative month; also, there were no significant differences when comparing cardiopulmonary bypass time and total operation time. Mean cross-clamping time was significantly shorter in the del Nido group (*P*<0.001).

**Conclusion:**

Del Nido cardioplegia may be a safe alternative to cold blood cardioplegia in adults undergoing cardiac surgical procedures with prolonged aortic cross-clamping time.

**Table:** Abbreviations, acronyms & symbols

ACC	= Aortic cross-clamping
AF	= Atrial fibrillation
ARE	= Aortic root enlargement
AVR	= Aortic valve replacement
BC	= Blood cardioplegia
BMI	= Body mass index
CABG	= Coronary artery bypass grafting
CBC	= Cold blood cardioplegia
CK-MB	= Creatine kinase myocardial band
COPD	= Chronic obstructive pulmonary disease
CPB	= Cardiopulmonary bypass
CVE	= Cerebrovascular event
DM	= Diabetes mellitus
DNC	= del Nido cardioplegia
EF	= Ejection fraction
ES	= Erythrocyte suspension
EuroSCORE	= European System for Cardiac Operative Risk Evaluation
GIS	= Gastrointestinal system
Hct	= Hematocrit
HL	= Hyperlipidemia
HT	= Hypertension
HTK	= Histidine-tryptophan-ketoglutarate
IABP	= Intra-aortic balloon pump
ICU	= Intensive care unit
LVEF	= Left ventricular ejection fraction
MI	= Myocardial infarction
MICS	= Minimally invasive cardiac surgery
MVR	= Mitral valve replacement
PVD	= Peripheral vascular disease
RV	= Right ventricular
TVA	= Tricuspid annuloplasty

## INTRODUCTION

Cardiopulmonary bypass (CPB) and cardiac arrest are indispensable components of cardiac surgery. Myocardial protection during cardiac surgery plays a key role in obtaining successful outcomes in cardiac surgical procedures. The goals of the myocardial protection during cardiac arrest are to prevent myocardial injury, to preserve myocardial function, and, at the same time, to provide a bloodless and motionless operating field^[1]^. Cardioplegia is an important component of intraoperative myocardial protection. Cardioplegia solutions induce a rapid and complete arrest of the heart, which is essential in many cardiac procedures. Many different methods and cardioplegia solutions have been introduced to ensure optimal myocardial protection. Nonetheless, the optimal cardioplegic solution for myocardial protection has not been determined until now^[1]^.

Cold blood cardioplegia (CBC) has been shown to preserve the myocardium effectively, however, it must be repeated every 15-20 minutes, prolonging aortic cross-clamping (ACC) and CPB times^[2,3]^. Single-dose cardioplegia strategy does not interfere with the flow of the surgery and was reported to shorten the CPB and ACC times. Histidine-tryptophan-ketoglutarate (HTK) is a single-dose intracellular cardioplegia which was introduced by Bretschneider in the 1970s. The safety and efficiency of a single dose of HTK in cardiac surgical procedures was shown in previous studies^[4]^. Although it is attractive, especially in long procedures, one single dose of HTK may lead to hemodilution and hyponatremia^[5]^. Besides, from a cost perspective, the HTK solution remains expensive compared to other cardioplegic solutions.

Del Nido cardioplegia (DNC) was developed by Pedro del Nido and his team in the 1990s for use in pediatric patients. DNC can be prepared by any in-house pharmacy, reducing costs^[6]^. Single-dose DNC solution has been used widely in congenital heart surgery for > 20 years and has been shown to be safe and effective in pediatric patients obtaining at least 90 minutes of cardiac arrest^[6]^. More recently, it has also been used in adult cardiac surgical procedures and was reported to provide comparable myocardial protection and clinical outcomes with reduced ACC time, CPB time, and operating time compared to standard cardioplegia strategies^[7-11]^. Yet, little randomized data exist, and its safety and efficiency in adult cardiac surgery need to be determined with large randomized controlled trials^[9-12]^. Furthermore, there is data scarcity for its safety and efficiency in patients with prolonged cross-clamping times. As far as we know, until now, only two retrospective studies examined its safety and efficiency in patients with prolonged ACC time^[13,14]^.

This study aims to investigate the efficacy and safety of DNC compared to CBC in patients with prolonged ACC time.

## METHODS

### Study Population and Design

This study was approved by our local Ethics Committee and complied with the requirements of the Declaration of Helsinki. We retrospectively reviewed the medical records of adult patients who underwent cardiac surgery under CPB between January 2014 and December 2019. One hundred consecutive adult patients undergoing cardiac surgical procedures using DNC with an ACC time ≥ 90 minutes were included in the study. One hundred consecutive adult patients undergoing cardiac surgical procedures with CBC who had an ACC time ≥ 90 minutes were the control group.

Patients who underwent emergency or salvage surgery, as well as reoperative surgery, patients with recent acute coronary syndrome, patients who required inotropes/vasopressor support in the preoperative period, patients who had dialysis-dependent renal failure, and patients who required a second period of ACC were excluded from the study. Patients who underwent arrhythmia surgery using cryoablation also were excluded due to its possible association with myocardial enzyme release.

### Operative Details

All procedures were performed using a standard general anesthesia protocol. Minimally invasive procedures were performed either with right infra-axillary minithoracotomy or ministernotomy under direct vision with central arterial and venous cannulation. The rest of the procedures were performed with a median sternotomy. Standard non-pulsatile CPB with a roller pump and a membrane oxygenator was used. In all procedures, the CPB circuit was shortened, and retrograde autologous priming was performed to reduce transfusion requirements^[15]^. The extracorporeal system was primed with a Ringer's lactate solution. CPB was established between the ascending aorta and the right atrium using dual-stage venous cannulation or bicaval cannulation. Prior to cannulation, 300-400 U/kg heparin sodium was administered to each patient to maintain activated clotting time values > 480 s. During CPB, the non-pulsatile pump flow was kept at 2.2-2.5 L/min/m^2^. The core temperature was cooled to 28°C. The alpha-stat strategy was used for pH management, and all patients were kept at normocapnic levels (PaCO_2_=35-45 mmHg). Concentrated fresh erythrocyte suspensions (≤ 7 days of storage) were added to the pump prime volume if required, to keep the hematocrit levels > 25% during CPB. The mean arterial pressure during CPB was stabilized between 50 mmHg and 70 mmHg.

### Cardioplegia Strategy

Myocardial protection was obtained with the administration of either CBC or DNC. No topical hypothermia was used. Antegrade cardioplegia was given at an aortic root pressure of 70-90 mmHg and delivered through the root cannula or by direct coronary ostial infusion. Retrograde cardioplegia was delivered at a pressure of 30-50 mmHg. A 500 mL of hot shot solution at 36°C was administered before the release of the aortic cross-clamp in all cases. The compositions of both cardioplegia solutions are detailed in Table 1. The hot shot solution was identical in composition to the maintenance dose of CBC.

**Table 1:** Composition of cardioplegia solutions.

	Del Nido Cardioplegia	Cold Blood Cardioplegia
Blood:crystalloid ratio	01:04	04:01
Base solution	1 L of Plasma-Lyte A	Saline (0.9% NaCl)
Mannitol	3.2 g/L	0
Magnesium sulphate	2 g/L	1 mg/mL (induction)
		0.5 mg/mL (maintenance)
NaHCO_3_	13 mEq/L	10 mEq/L
Potassium	26 mEq/L	30 mEq/L (induction)
		10 mEq/L (maintenance)
Lidocaine	130 mg	0

### Cold Blood Cardioplegia Strategy

CBC (1 L, induction) was given at a temperature of 4-8°C, and 500 mL of maintenance dose was repeated every 15-20 minutes. Combined antegrade-retrograde cardioplegia was delivered in patients who had coronary bypasses.

### Del Nido Cardioplegia Strategy

The heart was arrested with an initial induction dose of 20 ml/kg with a maximum dose of 1000 mL for patients > 50 kgs. Additional 500 mL was administered 60 minutes after the initial dose if the cross-clamping time was expected to be > 90 minutes. The induction and maintenance cardioplegia were delivered at 4°C. Cardioplegia was delivered anterogradely in all cases without any retrograde dosing.

### Primary Endpoints

The primary endpoints were clinical indicators for myocardial preservation, including postoperative peak troponin T levels, need for inotropic support, need for defibrillation after aortic cross-clamp removal, and postoperative left ventricular ejection fraction (LVEF) assessed by transthoracic echocardiography before discharge (average of five days, postoperatively) and at the sixth-month follow-up.

Cardiac troponin T levels were measured in the postoperative period at six, 12, 24, and 48 hours in both groups.

### Secondary Endpoints

Secondary endpoints were postoperative clinical outcomes, CBP and ACC times, and usage of blood products.

### Statistical Analysis

All statistical analyses were conducted using SPSS 22 software (IBM Corp. Released 2013. IBM SPSS Statistics for Windows, Version 22.0. Armonk, NY: IBM Corp.) All variables were investigated using visual (histograms, probability plots) and analytic (Kolmogorov-Smirnov test) methods to determine whether they were normally distributed. Continuous variables were reported as means and standard deviation for normally distributed variables and as medians and interquartile range for non-normally distributed variables. Categorical variables were presented using numbers and percentages. Comparison between the two groups was performed using the Chi-squared test or the Fisher's exact test for qualitative variables, the independent t-test for normally distributed continuous variables, and the Mann-Whitney U test for non-normally distributed continuous variables.

Propensity score matching analysis was performed to reduce the impact of selection bias and potential confounders secondary to non-randomization. The type of cardioplegic solution was used as a dependent binary variable, the following variables were included in a logistic regression model as independent variables to estimate the propensity scores: age, sex, body mass index, smoking, hypertension, diabetes mellitus, hyperlipidemia, chronic obstructive pulmonary disease, peripheral vascular disease, history of cerebrovascular events, European System for Cardiac Operative Risk Evaluation II, preoperative renal dysfunction, preoperative atrial fibrillation (AF), preoperative LVEF, preoperative hematocrit levels, and type of surgery. Cases with propensity scores differing by more than 0.05 were considered unmatched. Nearest neighbor matching without replacement was used to match DNC and CBC patients using linear propensity scores. Propensity matching identified 86 matched pairs for analysis. P-values < 0.05 were considered to indicate statistical significance.

## RESULTS

### Demographics and Baseline Clinical Profile

The clinical characteristics of the CBC (n=100; 51 males; median age 60 [53.2-68] years) and DNC (n=100; 46 males; median age 58 [49-66] years) groups are shown in Table 2. Propensity score matching yielded 88 DNC and 88 CBC patients. In both matched and unmatched cohorts, DNC and CBC groups were statistically similar in terms of demographics, comorbidities, preoperative baseline, and clinical characteristics (Table 2).

**Table 2:** Baseline characteristics and comorbidities.

	Unmatched			Matched		
	DNC (n=100)	CBC (n=100)	*P*-value	DNC (n=88)	CBC (n=88)	*P*-value
Age (years)	58 (49-66)	60 (53.2-68)	0.16	59 (52.25-67.75)	60 (51.25-67)	0.97
Male, n (%)	46 (46)	51 (51)	0.48	43 (48.9)	44 (50)	0.88
BMI (kg/m^2^)	24.5 (21.7-30.8)	26.4 (23.9-29.1)	0.11	24.45 (21.8-30.8)	25.7 (23.8-29)	0.26
Smoking, n (%)	34 (34)	34 (34)	1	30 (34.1)	32 (36.4)	0.75
HT, n (%)	36 (36)	39 (39)	0.66	32 (36.4)	34 (38.6)	0.76
HL, n (%)	18 (18)	24 (24)	0.3	18 (20.5)	19 (21.6)	0.85
DM, n (%)	26 (26)	20 (20)	0.3	19 (21.6)	20 (22.7)	0.85
COPD, n (%)	25 (25)	23 (23)	0.7	21 (23.9)	21 (23.9)	1
PVD, n (%)	5 (5)	6 (6)	0.75	5 (5.7)	6 (6.8)	0.75
History of CVE, n (%)	4 (4)	2 (2)	0.6	2 (2.3)	2 (2.3)	1
Renal dysfunction, n (%)	4 (4)	3 (3)	0.7	3 (3.4)	3 (3.4)	1
EuroSCORE II	2.06 (1.46-2.86)	1.81 (1.4-2.79)	0,61	2.06 (1.43-2.86)	1.91 (1.4-2.8)	0.89
Preoperative EF	55 (45-55)	55 (48-55)	0.26	55 (45.75-55)	55 (45-55)	0.2
Preop. AF, n (%)	12 (12)	13 (13)	0.8	11 (12.5)	7 (8)	0.32
Preop. Hct %	42 (40-46)	43 (40-47)	0.43	42 (40-46)	43 (40-47)	0.52

Values are presented as mean ± standard deviation, median (interquartile range), or n (%). *P*<0.05 was considered statistically significant AF=atrial fibrillation; BMI=body mass index; CBC=cold blood cardioplegia; COPD=chronic obstructive pulmonary disease; CVE=cerebrovascular event; DM=diabetes mellitus; DNC=del Nido cardioplegia; EF=ejection fraction; EuroSCORE=European System for Cardiac Operative Risk Evaluation; Hct=hematocrit; HL=hyperlipidemia; HT=hypertension; PVD=peripheral vascular disease

### Primary Endpoints in Matched Patients

There were no significant differences when comparing the two groups regarding the requirement for intraoperative defibrillation (10.2% *vs*. 15.9%; *P*=0.26), postoperative peak troponin T levels (478.7 ±33.4 *vs*. 490.9±53.1; *P*=0.07), perioperative inotropic support (12.5% *vs*. 17%; *P*=0.4), intra-aortic balloon pump (IABP) requirement (2.3% *vs*. 4.5%; 0.68), and postoperative LVEF at discharge (55 [45-55] *vs*. 50 [45-55]; *P*= 0.08) and on the postoperative sixth month (55 [48.7-55] *vs*. 50 [50-55]; *P*=0.34).

### Secondary Endpoints in Matched Patients

In the DNC group, mean ACC time (99 [94.2-106] *vs*. 124 [117-131]; *P*<0.001) was significantly shorter than in the CBC group. On the other hand, there were no significant differences when comparing the two groups regarding postoperative new AF development (21.6% *vs*. 29.5%; *P*=0.22), CPB time (161 [153.2-170.7] *vs*. 163.5 [156-175.5]; *P*=0.3), total operation time (245 [231.2-255] *vs*. 247.5 [240-255]; *P*=0.08), and transfusion rates (*P*=0.72).

There were no statistically significant differences in other intraoperative and postoperative clinical characteristics between the two groups in both matched and unmatched cohorts. The intraoperative and postoperative data of both groups are presented in Table 3 and Table 4, respectively.

**Table 3:** Operative characteristics.

Surgical procedures, n (%)	Unmatched			Matched		
	DNC (n=100)	CBC (n=100)	*P*-value	DNC (n=88)	CBC (n=88)	*P*-value
Single valve	31 (31)	32 (32)		27 (30.27)	22 (25)	
MVR	18 (18)	19 (19)		16 (18.2)	14 (15.9)	
AVR	2 (2)	3 (3)		2 (2.2)	2 (2.2)	
AVR + ARE	11 (11)	10 (10)		9 (10.2)	6 (6.8)	
Double valve	31 (31)	30 (30)		27 (30.27)	28 (31.8)	
Triple valve	7 (7)	8 (8)		7 (8)	8 (9.1)	
Valve surgery + CABG	9 (9)	7 (7)		7 (8)	7 (8)	
Aortic surgery	8 (8)	9 (9)		7 (8)	9 (10.2)	
Aortic surgery + valve surgery	4 (4)	6 (6)		4 (4.5)	6 (6.8)	
Aortic surgery + CABG	5 (5)	4 (4)		5 (5.7)	4 (4.5)	
Aortic surgery + CABG + valve surgery	5 (5)	4 (4)		4 (4.5)	4 (4.5)	
Total number of MICS	31 (31)	30 (30)	0.75	27 (30.7)	24 (27.3)	0.62
Minimally invasive MVR	18 (18)	19 (19)		16 (18.2)	14 (15.9)	
Minimally invasive AVR	1 (1)	1 (1)		1 (1.1)	1 (1.1)	
Minimally invasive AVR + ARE	4 (4)	5 (5)		4 (4.5)	4 (4.5)	
Minimally invasive double valve procedure (MVR + TVA)	8 (8)	5 (5)		6 (6.8)	5 (5.7)	
ACC time, min	99 (95-107.5)	123 (116.3-130.7)	< 0.001	99 (94.2-106)	124 (117-131)	< 0.001
Total CPB time, min	163 (154.2-171)	165 (156-174)	0.4	161 (153.2-170.7)	163.5 (156-175.5)	0.3
Total operation time, min	245 (235-255)	245 (236.2-255)	0.4	245 (231.2-255)	247.5 (240-255)	0.08
Defibrillation requirement, n (%)	9 (9)	15 (15)	0.19	9 (10.2)	14 (15.9)	0.26
Nadir Hct during CPB, %	25 (24-26)	25 (22-28)	0.15	25 (23.2-26)	25 (22-28)	0.44
Transfused ES, unit	1 (0-1)	0.5 (0-1)	0.3	0.5 (0-1)	1 (0-1)	0.72

Values are presented as mean ± standard deviation, median (interquartile range), or n (%). *P*<0.05 was considered statistically significant ACC=aortic cross-clamping; ARE=aortic root enlargement; AVR=aortic valve replacement; CABG=coronary artery bypass grafting; CBC=cold blood cardioplegia; CPB=cardiopulmonary bypass; DNC=del Nido cardioplegia; ES=erythrocyte suspension; Hct=hematocrit; MICS=minimally invasive cardiac surgery; MVR=mitral valve replacement; TVA=tricuspid annuloplasty

**Table 4:** Postoperative outcomes.

	Unmatched			Matched		
	DNC (n=100)	CBC (n=100)	*P*-value	DNC (n=88)	CBC (n=88)	*P*-value
Intubation time (hours)	8 (7-9.75)	9 (7-10)	0.14	8 (7-9)	9 (7-10)	0.11
ICU stay (days)	1 (1-2)	1 (1-2)	0.2	1 (1-2)	1 (1-2)	0.09
Hospital stay (days)	5 (5-6)	5 (5-6)	0.4	5 (5-6)	5 (5-6)	0.24
Peak troponin T (ng/L)	478.25 ± 32.01	486.38 ± 52.7	0.19	478.7 ±33.4	490.9±53.1	0.07
Inotropic support, n (%)	12 (12)	17 (17)	0.32	11 (12.5)	15 (17)	0.4
IABP requirement, n (%)	2 (2)	4 (4)	0.35	2 (2.3)	4 (4.5)	0.68
Perioperative MI, n (%)	0	1 (1)	1	0	1 (1.1)	1
CVE, n (%)	2 (2)	2 (2)	1	2 (2.3)	2 (2.3)	1
Respiratory failure requiring reintubation, n (%)	2 (2)	4 (4)	0.68	1 (1.1)	4 (4.5)	0.37
Pneumonia, n (%)	3 (3)	4 (4)	1	3 (3.4)	4 (4.5)	1
Postoperative new AF, n (%)	23 (23)	26 (26)	0.6	19 (21.6)	26 (29.5)	0.22
Re-exploration for bleeding, n (%)	2 (2)	3 (3)	1	1 (1.1)	3 (3.4)	0.62
Mediastinitis, n (%)	1 (1)	1 (1)	1	0	0	1
Wound infection, n (%)	6 (6)	5 (5)	0.75	4 (4.5)	5 (5.7)	1
Acute renal dysfunction, n (%)	4 (4)	5 (5)	1	4 (4.5)	5 (5.7)	1
GIS complications, n (%)	0	0		0	0	
EF before discharge	50 (45-55)	50 (45-55)	0.14	55 (45-55)	50 (45-55)	0.08
EF by the sixth postoperative month	55 (45-55)	50 (50-55)	0.37	55 (48.7-55)	50 (50-55)	0.34
RV dysfunction, n (%)	0	0	1	0	0	1
In-hospital mortality, n (%)	3 (3)	3 (3)	1	2 (2.3)	3 (3.4)	1

Values are presented as mean ± standard deviation, median (interquartile range), or n (%). P<0.05 was considered statistically significant AF=atrial fibrillation; CBC=cold blood cardioplegia; CVE=cerebrovascular event; DNC=del Nido cardioplegia; EF=ejection fraction; GIS=gastrointestinal system; IABP=intra-aortic balloon pump; ICU=intensive care unit; MI=myocardial infarction; RV=right ventricular

## DISCUSSION

In the present study, we have found that there were no significant differences when comparing the two groups regarding the requirement for intraoperative defibrillation, postoperative peak troponin T levels, perioperative inotropic support, IABP requirement, and postoperative LVEF at discharge and on the sixth postoperative month. Furthermore, we have found that DNC was associated with reduced ACC compared to the CBC group. There were no statistically significant differences regarding CPB time and total operation time between the two groups. Compared with CBC, DNC provided similar outcomes and comparable myocardial protection in patients that required prolonged ACC time. Our findings were similar to the results of previous studies^[13,14]^.

Although previous studies demonstrated non-inferior or better myocardial protection in various cardiac surgical procedures with the use of DNC^[9-13]^, only a few randomized controlled trials examined the safety of DNC. In a randomized study by Ad et al.^[9]^, DNC was compared with blood cardioplegia (BC) in patients undergoing isolated coronary artery bypass grafting (CABG). Defibrillation rates were similar in both groups. DNC group had shorter CPB (97 *vs*. 103 minutes) and ACC (70 *vs*. 83 minutes) times, yet there was no significant difference between the DNC group and the control group. Although not significant, postoperative troponin levels were lower in the DNC group. It was shown that DNC could be used as an alternative to BC solution in adults with comparable clinical outcomes and improved surgical workflow^[9]^. Sanetra et al.^[11]^ compared DNC with BC in a randomized trial in patients undergoing aortic valve replacement. The postoperative troponin and creatine kinase myocardial band (CK-MB) levels were lower in the DNC group, but statistical significance was not seen. ACC time (55.2 *vs*. 55.5 minutes) and CPB time (67.9 *vs*. 69.2 minutes) were also similar. The defibrillation requirement was significantly lower in the DNC group. They found that both groups had similar clinical outcomes and reported that DNC was an acceptable alternative for CBC in patients undergoing aortic valve replacement^[11]^. Ucak et al.^[10]^ compared DNC with intermittent warm BC in patients undergoing CABG. They demonstrated that DNC was associated with shorter ACC times (43.7 *vs*. 54.3 minutes) and CPB times (67.9 *vs*. 77.2 minutes). There was no difference in defibrillation requirement and clinical results between the two methods, including postoperative myocardial enzyme release^[10]^. Mehrabanian et al.^[12]^ compared DNC with HTK in adult patients undergoing combined cardiac surgery. There was no difference in postoperative myocardial enzyme release, postoperative ejection fraction, ACC time, CPB time, perioperative transfusions, and clinical outcomes between the two groups^[12]^.

Although many studies have been reported on the efficiency and safety of DNC in adult cardiac surgery, data on safety and efficiency of DNC in complex adult cardiac procedures with prolonged cross-clamping times are scarce. Kim et al.^[14]^ investigated DNC's safety and efficiency in adults undergoing multiple and complex cardiac surgical procedures. Peak levels of postoperative cardiac enzymes were lower in the DNC group but without statistical significance. DNC group had shorter ACC (76.1 *vs*. 94.8 minutes) and CPB (98.6 *vs*. 148.4 minutes) times. They reported comparable myocardial protection and postoperative clinical outcomes with DNC compared to BC in procedures with mean cross-clamping times < 95 minutes^[14]^. Lenoir et al.^[13]^ investigated DNC's efficiency in adult patients undergoing aortic root surgery with mean cross-clamping time > 140 mins. DNC was associated with shorter ACC (145 *vs*. 161 minutes) and CPB (163 *vs*. 181 minutes) times compared to the BC group. Troponin T and CK-MB levels were similar in both groups. DNC group was found to be associated with more frequent return to spontaneous sinus rhythm. They reported comparable postoperative clinical outcomes compared to BC^[13]^.

While providing appropriate myocardial protection is indispensable, reducing ACC and CPB times is also important to improve the perioperative outcomes^[16,17]^. Single shot of cardioplegia was reported to reduce ACC, CPB, and operative times while improving surgical flow^[13,14]^. Each additional cardioplegia dosing disrupts the surgical flow and potentially increases ACC, CPB, and operative times, notably in patients who receive multidose cardioplegia. We hypothesized that in patients undergoing complex cardiac surgical procedures, with a single dose of DNC cardioplegia, duration of ACC, CPB, and operative times would be significantly reduced compared to multidose cardioplegia, and this would translate into a positive effect on patients' clinical outcomes. We have found that DNC was associated with reduced ACC compared to the BC group. However, unlike previous studies, in the current study, there were no statistically significant differences regarding CPB time and total operation time despite shorter ACC. Lenoir et al.^[13]^ and Kim et al.^[14]^ reported reduced CPB and ACC times with the use of DNC in patients with prolonged ACC. It may be argued that similar CPB time despite shorter ACC in the DNC group is related to insufficient myocardial protection. Other clinical indicators of myocardial protection suggest the non-inferiority of DNC in our study. Although not statistically significant, peak troponin levels, defibrillation rate, inotropic support rate, IABP support rate, and postoperative new AF rate were lower in the DNC group. Therefore, we think that similar CPB time despite shorter ACC in the DNC group may be due to the delayed recovery of ventricular function related to the residual effect of lidocaine after aortic cross-clamp removal that we observed in our clinical practice as reported in previous studies. Govindapillai et al.^[18]^ examined if lidocaine in DNC is adequate to prevent sodium influx via the window current in isolated rat hearts. In their study, they observed that the time to the return of the first heartbeat in the DNC was twice as long compared to BC, similarly to their experience in their clinical practice after switching from standard to DNC for their pediatric patients. They suggested that persistent inactivity during early reperfusion may improve myocardial recovery in a manner similar to that seen with the use of warm terminal cardioplegia^[18]^. Vlooran et al.^[19]^ also reported delayed recovery of the heart after aortic declamping with the use of DNC. Delayed recovery was attributed to the residual effect of lidocaine, and it was suggested that delayed recovery might help to improve myocardial recovery in a similar way seen with the use of warm terminal cardioplegia. O'Blenes et al.^[20]^ investigated the ability of DNC to protect aged cardiomyocytes during cardioplegic arrest and reperfusion in rats. They reported that in cardiomyocytes arrested with DNC, contraction amplitude during early reperfusion was significantly lower, and return to baseline was delayed.

In the present study, postoperative peak troponin T levels were similar in both groups. Likewise, Lenoir et al.^[13]^ reported similar myocardial injury with DNC and BC in clamping times < 180 minutes. But, beyond 180 minutes of ischemia, they reported an abrupt increase in the postoperative release of myocardial biomarkers in the DNC group, suggesting suboptimal myocardial protection in this subgroup^[13]^. Mick et al.^[21]^ compared DNC and Buckberg cardioplegia in isolated valve procedures. Troponin T levels did not differ statistically in the two groups, yet longer ACC times were associated with higher troponin T levels in both groups, suggesting impaired myocardial protection with extended periods of ischemia. Despite the low cross-clamping time in the DNC, it may be argued that CBC protects the heart better due to similar troponin levels and clinical outcomes in both groups. We think that this conclusion cannot be drawn from our study. Although not statistically significant, peak troponin levels, defibrillation rate, inotropic support rate, IABP support rate, and postoperative new AF rate were lower in the DNC group.

Perioperative transfusions in patients undergoing cardiac surgery have been found to be associated with increased morbidity and mortality^[22]^. In the present study, transfusion rates were similar between the two groups. Ad et al.^[9]^ have shown that patients in the DNC group had similar transfusion rates compared to the CBC group. In patients with prolonged cross-clamping times, as numbers of additional doses of cardioplegia increase, mixed crystalloid/blood cardioplegia can potentially lead to marked hemodilution and increase transfusion requirements. Sanetra et al.^[11]^ found no difference in transfusion requirement between the DNC and BC (4:1 BC) group. Lenoir et al.^[13]^ also could not find a difference in transfusion rates between DNC and BC (blood:crystalloid ratio is 4:1) groups in patients with prolonged ACC. On the other hand, Kim et al.^[14]^ found that transfusion rates were lower in the DNC group than in the BC group (blood:crystalloid ratio is 4:1) in patients with prolonged ACC.

### Limitations

This study has several limitations. This is a single-center retrospective study; thus, randomization and blinding were not possible. Although our patients had prolonged ACC time, they were relatively low-risk patients. Hence, our results cannot be generalized to high-risk patient populations. We compared DNC with CBC. Thus, our results may not be generalizable to modifications of DNC and other cardioplegic solutions. ACC time was not included as a variable in the propensity-matched analysis. In patients undergoing similar surgical procedures, the ACC time will be shorter in those who receive single-shot cardioplegia, compared to multidose cardioplegia strategies, particularly in patients with prolonged cross-clamping time. Thus, if both groups had similar cross-clamping time, DNC group would include patients undergoing more complex procedures.

## CONCLUSION

DNC may be a safe alternative to CBC in adults undergoing cardiac surgical procedures with prolonged ACC time. Nevertheless, our results may not be generalizable to modifications of DNC and other cardioplegic solutions.
